# The Death-Rate from Phthisis

**Published:** 1911-11-04

**Authors:** 


### THE DEATH-RATE FROM PHTHISIS.

The cherished theory that the whole of the fall in the death-rate from phthisis can be attributed to improved sanitary conditions receives somewhat trenchant criticism in a pamphlet by Prof. Karl Pearson, issued recently from the Eugenics Laboratory (Dulau & Co., price 1s.). The subject is one of first-class importance at the present time, and in view of the impetus which is now being given to the building of sanatoria and of the imminent intervention of the State in the fight against tuberculosis, it is well that the question in all its bearings should be dispassionately considered. We therefore welcome the appearance of this keenly critical pamphlet, which should effectively counteract some of the extravagant claims which have been freely made by many of the best authorities regarding the results of contemporary methods in dealing with phthisis. Prof. Karl Pearson demonstrates clearly from the statistics at his command that the death-rate from phthisis during the period of our supposed greatest knowledge and during the time of our most active campaign against tuberculosis has ceased to be reduced at the old rate, and that there is an actual slackening in the rate of fall. The carefully plotted curves of the diagrams do not continue their fall but tend to become parallel to the horizontal base-line. Consideration of the figures giving the deaths from phthisis as a percentage of all deaths, leads to the conclusion that this percentage of deaths from phthisis has now become stationary, or is even slightly rising. These facts will be cold comfort to sanatorium and dispensary enthusiasts, but they will have to be faced.

Prof. Pearson has some hard things to say in criticism of Dr. Newsholme's book, "The Prevention of Tuberculosis." He calls attention to the absence of reliable evidence as to the efficiency of sanatoria as far as the cure of tuberculosis is concerned. But is it not a fact that the public has been appealed to on the grounds that sanatoria are places intended for the *cure* of tuberculosis, and not on the value of these institutions as leper houses—that is to say, as asylums? It is high time that this essential difference between hospitals for consumptives and sanatoria should be made clear. For the incurable phthisical individual—and unfortunately there are many such—the sanatorium does not offer a home or a hope. Such a person needs an institution designed on the Norwegian system, and no amount of enthusiasm can make us blind to the fact, so well demonstrated by recent German statistics, that it is unfair to the community to admit into the ordinary sanatorium, which is essentially a hospital, a patient who has little chance of being cured by the treatment, though his condition may be improved.

A thorough reconsideration of the position cannot fail to be beneficial, and a resifting of the evidence by others may prevent the launching of unprofitable schemes before it is too late. It is difficult to reconcile the fall in the phthisis death-rate before 1866 and the *retardation* in fall since 1895 with any hypothesis that the fall is the result of increased segregation of the tuberculous, of improved environment, or of any change of treatment. How is it, asks Prof. Karl Pearson, the phthisis death-rate fell long before the general death-rate, and why has the intensified campaign against tuberculosis been accompanied by not a quickened but by a retarded rate of fall, while the general death-rate itself has for the same period shown a quickened fall? The explanation offered is the occurrence of that factor so well known to epidemiologists—namely, the ratio of the number of non-immune individuals to the population at large. If the selection due to many years of heavy phthisis mortality has left us with a more immune and resistant population, then it must be admitted that *infection* is not the only factor to be considered. There remains to be seriously reckoned with the question of hereditary immunity. Prof. Pearson draws attention to the data showing that a very large majority of persons do not escape infection by the tubercle bacillus, and that the bulk of the population has sufficient resistive power to survive the attack and eventually dies from other causes. It must be admitted that the prevalence of non-lethal tuberculosis is exceeding widespread, and the impression becomes strong that the constitutional factor in this matter must be a very important one; the investigations made in the Eugenics Laboratory point to the conclusion that, in the main and on the average of large numbers, constitution is a question of inheritance. Of course the suggestion is that natural selection may have done more for health in this matter than medical science, but however this may be, this latest issue from the Department of Applied Statistics of University College is worthy of careful study and should in its turn provoke authoritative criticism.

